# Quantitative Trait Loci for Bone Lengths on Chromosome 5 Using Dual Energy X-Ray Absorptiometry Imaging in the Twins UK Cohort

**DOI:** 10.1371/journal.pone.0001752

**Published:** 2008-03-12

**Authors:** Usha Chinappen-Horsley, Glen M. Blake, Ignac Fogelman, Bernet Kato, Kourosh R. Ahmadi, Tim D. Spector

**Affiliations:** 1 Twin Research and Genetic Epidemiology Unit, King's College London School of Medicine, St Thomas' Hospital, London, United Kingdom; 2 Department of Nuclear Medicine, King's College London School of Medicine, Guy's Hospital, London, United Kingdom; The University of Queensland, Australia

## Abstract

Human height is a highly heritable and complex trait but finding important genes has proven more difficult than expected. One reason might be the composite measure of height which may add heterogeneity and noise. The aim of this study was to conduct a genome-wide linkage scan to identify quantitative trait loci (QTL) for lengths of spine, femur, tibia, humerus and radius. These were investigated as alternative measures for height in a large, population–based twin sample with the potential to find genes underlying bone size and bone diseases. 3,782 normal Caucasian females, 18–80 years old, with whole body dual energy X-ray absorptiometry (DXA) images were used. A novel and reproducible method, linear pixel count (LPC) was used to measure skeletal sizes on DXA images. Intraclass correlations and heritability estimates were calculated for lengths of spine, femur, tibia, humerus and radius on monozygotic (MZ; n = 1,157) and dizygotic (DZ; n = 2,594) twins. A genome-wide linkage scan was performed on 2000 DZ twin subjects. All skeletal sites excluding spine were highly correlated. Intraclass correlations showed results for MZ twins to be significantly higher than DZ twins for all traits. Heritability results were as follows: spine, 66%; femur, 73%; tibia, 65%; humerus, 57%; radius, 68%. Results showed reliable evidence of highly suggestive linkage on chromosome 5 for spine (LOD score  =  3.0) and suggestive linkage for femur (LOD score  =  2.19) in the regions of 105cM and 155cM respectively. We have shown strong heritability of all skeletal sizes measured in this study and provide preliminary evidence that spine length is linked to the chromosomal region 5q15-5q23.1. Bone size phenotype appears to be more useful than traditional height measures to uncover novel genes. Replication and further fine mapping of this region is ongoing to determine potential genes influencing bone size and diseases affecting bone.

## Introduction

Human height is a highly heritable and complex trait. Heritability estimates measured in previous studies range from 0.68–0.98 (with men having higher heritability than women) [1]; [2]. The polygenic and heterogeneic nature of height means that it may be difficult to find causal genes and explains the lack of success to date in previous studies despite large numbers [3]–[6].

Dividing height into sub-components may improve the search for height genes. Previous studies have shown demi-span and lower limb length as useful surrogates for height [7]–[10]. In a Latin American sample, knee height was found to be a good surrogate for height in elderly populations and highlighted ethnic-specific height predictor equations are required to account for the differences between populations [10]. Furthermore, sub-compartments of bone and skeletal measures are highly heritable [10]–[13], for example, hip axis length with a heritability of 62% [11].

The aim of this study was to conduct a genome-wide linkage scan to identify quantitative trait loci (QTL) for lengths of spine, femur, tibia, humerus and radius as alternatives for height in a large, population–based twin sample and to identify the genes for height which may also be underlying diseases affecting bone size.

## Results

Our results describe a normal population of 3,751 Caucasian females. The summary statistics comparing identical and non-identical Caucasian females are shown in Table 1. Both sub-groups are similar in all variables and constitute a healthy population with an average BMI of approximately 25kg/m^2^. The mean age of MZ individuals was approximately 2 years older than that of DZ individuals.

**Table 1:** Twin descriptives

	MZ *(n = 1,157)*			DZ *(n = 2,594)*		
	Range	Mean	SD	Range	Mean	SD
**Age (yrs)**	17–80	50.58	14.00	17–79	48.10	13.10
**Height (cm)**	144–180.5	161.35	6.22	141.5–183	162.29	6.12
**Weight (kg)**	36.7–125	66.50	12.33	35.6–128.3	67.50	12.64
**BMI (kg/m** ^2^ **)**	15.1–52.7	25.58	4.76	13.9–51.4	25.64	4.70
**Spine Length (cm)**	35.1–49.4	42.30	2.34	32.5–50.7	42.59	2.32
**Femur Length (cm)**	37.7–49.4	42.74	2.12	35.1–49.4	42.97	2.13
**Tibia Length(cm)**	29.9–41.6	35.80	2.05	29.9–41.6	36.01	1.99
**Humerus Length (cm)**	21.4–34.1	28.58	1.71	22.3–34.0	28.78	1.71
**Radius Length (cm)**	22.1–28.7	25.29	1.32	21.8–28.6	25.32	1.37

Length of femur, tibia, humerus and radius were highly and significantly correlated with a value of 0.63 or above with all remaining variables with the exception of spine, between 0.20–0.25 and the correlation between lengths of radius and humerus at 0.49 (Table 2).

**Table 2:** Correlation matrix between lengths

	SPINE LENGTH	FEMUR LENGTH	TIBIA LENGTH	HUMERUS LENGTH
**FEMUR LENGTH**	0.246	-	-	-
**TIBIA LENGTH**	0.223	0.777	-	-
**HUMERUS LENGTH**	0.204	0.688	0.641	-
**RADIUS LENGTH**	0.233	0.647	0.672	0.493

Heritability is shown in Table 3 along with 95% confidence intervals for each bone length and intra-class correlations (ICC). MZ twins had significantly higher ICC than DZ twins and the highest heritability was found for length of femur at 73%.

**Table 3:** Heritability of Bone Lengths

Phenotype	ICC		h^2^ (AE model)
	MZ	DZ	
Spine Length	0.66	0.31	0.65 [0.58–0.71]
Femur Length	0.85	0.48	0.73 [0.57–0.89]
Tibia Length	0.81	0.48	0.65 [0.49–0.81]
Humerus Length	0.71	0.42	0.57 [0.39–0.74]
Radius Length	0.66	0.36	0.68 [0.62–0.74]

* ICC  =  Intraclass correlations

**Figure 1:**
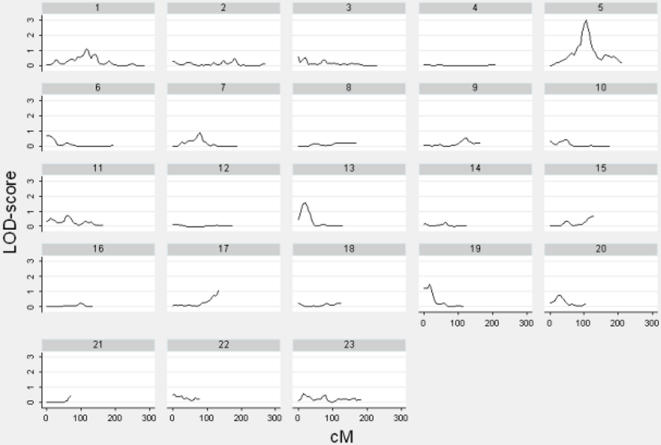
Linkage Results (LOD-score vs. cM) for Spine Length Residuals. LOD-scores are illustrated for chromosomes 1–23 for spine length residuals. There is one highly suggestive peak (LOD  =  3.00) on chromosome 5.

**Figure 2:**
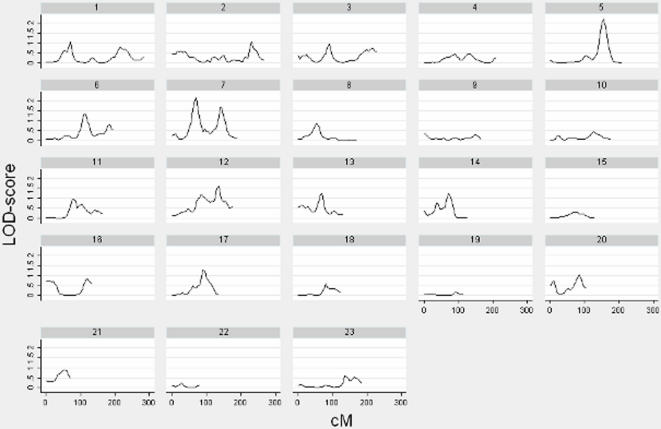
Linkage Results (LOD-score vs. cM) for Femur Length. LOD-scores are illustrated for chromosomes 1–23 for femur length. There is one suggestive peak (LOD  =  2.19) on chromosome 5.

**Figure 3:**
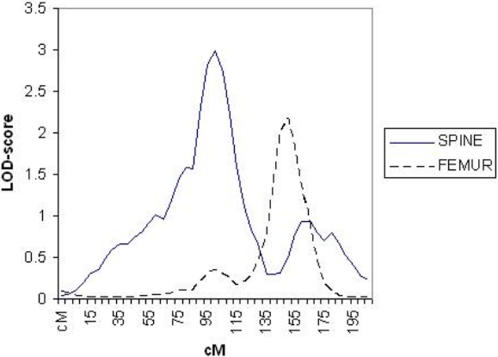
Linkage Results for Spine & Femur on Chromosome 5 at 105cM and 155cM respectively. LOD-scores are compared on chromosome 5 for spine length residuals (LOD = 3.00) and femur length (LOD = 2.19) at 105cM and 155cM respectively.

Multipoint linkage analysis was performed on all bone lengths but only two sites showed suggestive results. Figures 1 and 2 illustrate the LOD-score peaks on chromosome 5 for both spine and femur. Figure 3 compares LOD scores for spine (LOD = 3.00) and femur (LOD = 2.19) at 105cM and 155cM respectively on chromosome 5.

As a confirmation of the results, empirical p-values were obtained using a permutation approach. The results for each phenotype are shown in Table 4. Using National Centre for Biotechnology Information MapViewer (NCBI, National Library of Medicine, National Institute of Health, MD 20894, USA), this region was located at 5q15-5q23.1 and the nearest marker was found to be WIAF-1594 at marker location 106.99cM.

**Table 4:** Highly Suggestive/Suggestive Multipoint LOD-scores on Chromosome 5

Phenotype	Nominal LOD-score	Nominal P-value	Empirical p-value
Spine (n = 927)	3.00	0.0001	0.0002
Femur (n = 968)	2.19	0.0002	0.0004

## Discussion

Our main findings showed that all bone sizes measured were highly heritable. Moreover, the length of spine had a highly suggestive LOD-score after linkage analysis at the region 5q15-5q23.1.

There are few studies which have previously reported linkage results on chromosome 5 using traditional height measures. Deng et al, 2002 [12] used 53 European-American human pedigrees to show a maximum LOD score of 2.14 in multipoint linkage analysis at the region 5q31 with the peak marker D5S2115. A slightly higher LOD score of 2.26 and a different peak marker, D5S816, was also found in the region 5q31 by Wu et al, 2003 [13] using 1100 European-American individuals. Lastly, Willemson et al, 2004 [14] studied 174 Dutch families resulting in a LOD score of 2.04 at 5p14.3-p13.3 with markers D5S2845-D5S1470.

A more recent and larger study by Perola et al, 2007 [4] used genotypic data from the GenomEUtwin consortium which consists of eight twin cohorts of European origin. Genome-wide scans were performed for 3,817 families (8,450 individuals) taken from Australian, Danish, Finnish, Dutch, Swedish and United Kingdom twin cohorts. Age, sex and country of origin were used as covariates in the variance component linkage analysis. Heritability for height was 81% and there was evidence for a significant QTL of 3.28 on chromosome 8 at the region 8q21.3. Suggestive QTLs were also found on chromosomes Xq25 (LOD = 2.03), 7p22.3 (LOD = 2.03) and 20p13 (LOD = 1.4–1.69) but none overlapping our regions. This study suggests that a greater number of individuals may be needed to find the genes for height than for bone size.

An earlier smaller study by Perola et al, 2001 [6] also investigated height in five study groups, comprising of 614 individuals from 247 families, mostly from the Finnish Twin Cohort and representing a population-wide sample. Each of the five genome scans had approximately 350 evenly spaced markers genotyped on 22 autosomes. A variance-component method was used to analyze the genotype data and results showed a maximum multipoint LOD score of 2.91 at chromosome 7pter for height and a second locus with a suggestive maximum multipoint LOD score of 2.61 at chromosome 9q. Chromosome 7 was also supported by the data by Hirschhorn et al [5] who used a similar method.

In our study, age was responsible for 11% of the variation in spine length which is why it may be useful to stratify the sample population into age groups. Age-stratification was applied to a subset of the Framingham Heart Study [15] where linkage analyses were contrasted for height among other variables. The data was categorized into three age groups: 31–49, 50–60, 61–79. Genome-wide QTL analyses were performed using Sequential Oligogenic Linkage Analysis Routines (SOLAR, Southwest Foundation for Biomedical Research, Texas, USA). A linkage signal for height was detected on chromosome 14q11.2 near marker GATA74E02A (LOD  =  2.38, ages 31–49, LOD  =  1.84, ages 50–60, LOD  =  2.45, ages 61–79). The age-stratified results suggest that QTLs expressed over long periods of time and affecting multiple, correlated traits may be identified using genome scans and variance-component analysis to help detect early and/or late gene expression.

Bioinformatic investigation showed our peak marker to be WIAF-1594 at a LOD-score of 3.00 for the spine in the region 5q15-5q23.1. This marker codes for a key enzyme controlling cellular oxidative stress-glutaredoxin (thioltransferase). It works in catalyzing the reduction of glutathionyl protein disulfide bonds [16]. Another study has shown the importance of thioltransferase in maintaining mitochondrial function in the central nervous system (CNS) on studies involving mitochondrial dysfunction in the lumbo-sacral cord [17].

This chromosomal region has also been linked to kyphoscoliosis on chromosomal region 5q13 in a small study of seven families [18]. This condition is characterised by a lateral spinal curvature in conjunction with a thoracic kyphosis in excess of the normal range. Candidate loci in this region include IRX genes which code for homeobox proteins associated with embryonic midline development. Hence, the genes needed for spinal development and enzymes required for maintenance of the spinal cord may be associated with adult spine length.

Finally, this same region has been previously implicated in certain rare, monogenic diseases yet may overlap in those which are more common and widespread. There is a well-defined syndrome called 5p-, whose characteristics include short stature, where there is an interstitial deletion in the 5q13.1-q15 region. Krishna et al, 1997 [19] hypothesised that the short stature may possibly be due to growth hormone deficiency as the growth hormone receptor gene (GHR) gene resides at 5p14-p12 and in addition, there are several genes for growth factors and growth factor receptors present on 5q.

A major limitation of this study was the sole use of women. It has been widely established that there are significant gender differences in bone geometry where the genes for men may be different to those of women [20]. Men are generally larger in skeletal proportions compared to women which may be a fundamental reason why more fractures occur in women after peak bone mass is acquired.

Another potential limitation in this study is that the sample population is twin-based. However, with regards to zygosity, it has been shown by Andrew et al, 2001 [21] that twins are generaliseable to singletons for many common traits including bone mineral density, osteoarthritis, lipids and blood pressure. Therefore, this study may be applied to surrogate measures for height in the general population. Nevertheless, there remains some controversy as to whether birthweight affects adult skeletal size and in a twin population, MZs are generally slightly lighter in weight than DZs. Previous studies have shown this defining anthropometric measure to be associated with bone mineral content [22] so there is a possibility that it may also be associated with bone size. However, we do not believe these small differences at birth will have altered our QTLs of interest and believe the results will be generalisable.

Finally, a potential drawback of linkage pronged analysis is one of power. Many recent publications using genome-wide association scans have highlighted that the genetic variants which underpin height variation as well many other complex traits are likely to have very small effect sizes [23]–[25]. For example, Weedon et al (2007) explained that typical effect sizes for variants associated with height are likely to be extremely small and subsequently, linkage analysis would have little power to identify these variants mixture unless thousands of families were included in the study. While we generally agree with this conclusion we would like to emphasise that we currently know very little about the validity of the common disease/common variant model for all genetic variants that underlie common, complex traits. The true model is likely to include both rare and common variants with variable penetrance manifesting their effects in particular environments. Thus, if allelic heterogeneity is widespread for loci associated with complex traits–i.e. several variants within the same region/gene are associated with the same phenotype–or that variation in complex phenotypes such as height are also due to numerous relatively rare loci indirect, linkage disequilibrium-based association studies would have very limited power and linkage-based gene mapping techniques would be the preferred approach. Indeed, our aim for future studies include utilising genome-wide linkage and association analysis to which the twin design is easily amenable.

### Conclusion

Height has a higher variation amongst the general population than other bone measures such as femur or tibia length. This is mainly due to spine variation which can be due to a number of reasons, eg. spinal degeneration, osteoporosis or scoliosis. Height undoubtedly changes more dramatically than other bone sites after attainment of peak bone mass. For this reason, it is important to investigate alternative skeletal sites which are not affected by age or disease-related. We believe that alternative and more specific measures of skeletal size may indeed offer considerable advantages over classical height studies for gene discovery.

In conclusion, we have shown that variations in bone lengths are highly heritable and we report suggestive, preliminary evidence that spine length is linked to the region 5q15-5q23.1. Replication and further fine mapping is ongoing to identify genes that may influence bone length which may be associated with the same genetic regions coding for diseases affecting bone size.

## Materials and Methods

### Sample and Phenotypes

The Twins UK Adult Twin Registry provided the data for this study [26]; [27]. All participants gave written informed consent before entering the study and the St Thomas' Hospital research ethics committee approved the project.

**Figure 4:**
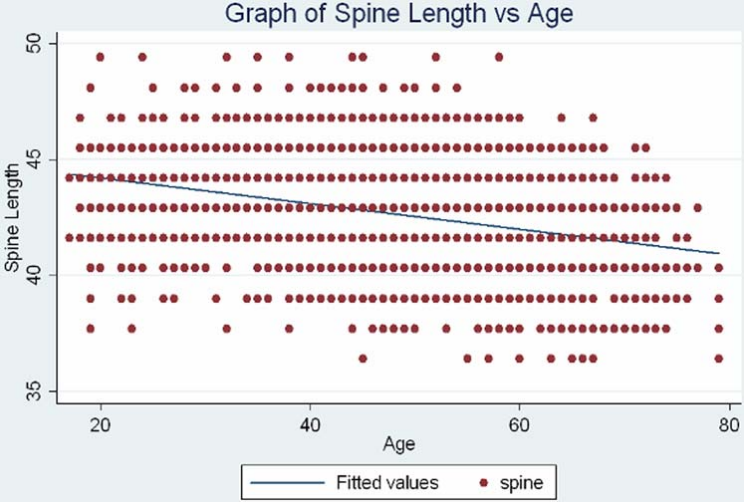
Graph of Spine Length vs. Age (showing line of best fit). The relationship between spine length and age is shown. Age accounted for 11% of the variation in spine length affecting the distribution in our sample. This variation was reduced to 8% after subjects over 70 years of age were removed.

The study used 3,782 normal Caucasian females, 18–80 years old, with whole body dual energy x-ray absorptiometry (DXA) data although the age was restricted to 70 years of age to exclude degenerative changes in analysis of the spine length measurement. Spine was also age-adjusted and residuals were used since age accounted for 11% of the variation in spine length affecting the distribution in our sample. This variation was reduced to 8% after subjects over 70 years of age were removed. Figure 4 illustrates the relationship between age and spine length.

### Linear Pixel Count (LPC) Method

A sub-sample of 90 subjects was first used to confirm the reproducibility and validity of a novel measurement technique in DXA total body analysis–linear pixel count (LPC). This method was reproducible with a mean coefficient of variation (CV%) of 1.6% among all bone size phenotypes measured. It was also validated against real clinical measures (X-rays and anthropometry) showing positive correlation & a relatively low population CV% [28].

LPC uses the regions of interest (ROI) sub-regional analysis mode on a Hologic QDR-4500W DXA scanner (Hologic Inc., Bedford, MA) to determine lengths of bones. The phenotypic measurements were of length of spine (C4 to L4), femur (greater trochanter to medial condyle), tibia (medial condyle to medial malleolus, humerus (head of humerus to medial epicondyle) and radius (olecranon process of ulna to ulna styloid process).

### Statistical analysis

A multipoint genome-wide linkage scan using 400 microsatellite markers spaced approximately every 10cM (with a surplus of 337 markers) was performed using statistical package, STATA v.9.0 (StataCorp) [29] using 1000 DZ female twin pairs with phenotypic information on bone lengths and ratios between bone lengths using robust regression analysis. Intra-class correlations investigating the ratio of the variance between and within pairs for 1,157 monozygotic (MZ) and 2,594 dizygotic (DZ) twins for height, length of spine, femur, tibia, humerus and radius were calculated in STATA.

Estimates for heritability were then calculated on the same bone sites using the structural equation modelling package, Mx [30]. In highly heritable phenotypes, above 60%, a genome-wide scan was implemented using a modified version of the Haseman and Elston method [31] in a generalised linear model in which the square of the sibling differences was regressed on estimated identical-by-descent (IBD) status at each locus [32]. Significant results were confirmed by computing empirical p-values for each LOD-score using a permutation approach [33]. Chromosomal regions were prioritised if the LOD score was greater than 2.

### Genotyping

Standard fluorescence-based genotyping methodologies were used and these have been described in detail in previous papers [34].
